# Role of TRPV1 in neuroendocrine regulation: a potential target against obesity?

**DOI:** 10.3389/fimmu.2025.1598804

**Published:** 2025-07-03

**Authors:** Jiexin Wang, Maohui Liu, Lingmiao Wen, Pengfei Xing, Jiawei Chen, Xiuwen Xia, WeiJun Ding

**Affiliations:** School of Basic Medical Sciences, Chengdu University of Traditional Chinese Medicine, Chengdu, China

**Keywords:** transient receptor potential vanilloid subtype 1 (TRPV1), obesity, neuromodulatory mechanisms, endocrine mechanisms, energy metabolism

## Abstract

Obesity is a common metabolic syndrome in which an imbalance between energy intake and consumption is the main cause of excessive accumulation of body fat. The increasing prevalence of obesity and its associated complications poses significant challenges to public health. Activation of the transient receptor potential vanilloid subtype 1 (TRPV1) cascade plays a key role in lipid metabolism and energy intake. TRPV1 is expressed across the central nervous system and peripheral organs is involved in the regulation of hormone secretion, appetite and mitochondrial function, and is recognized as one of the key targets for preventing obesity. The current treatments for obesity exhibit limited efficacy and are associated with numerous side effects. Targeting TRPV1 represents a potentially effective approach for managing obesity. In this work, by combining the recent mechanism of the role of TRPV1 in neuroendocrine regulation, we hope to provide novel approaches to block or even reverse the development of obesity.

## 1 Introduction

The increasing prevalence of obesity is expected to affect 4 billion people by 2035 (1–3). As a common metabolic syndrome, an imbalance in energy storage and expenditure is the main cause of excessive accumulation of body fat (4–7). Recent studies have shown that neuroendocrine modulation plays an important role in regulating adipose tissue thermogenesis and lipid metabolism (8). Body weight homeostasis is regulated by the coordinated interactions of nutrients, circulating neuroendocrine hormones, the central nervous system, and peripheral nerves, and even the release of hormonal signals from endocrine tissues is largely regulated by the peripheral nervous system (PNS) (9–13). Sympathetic nervous system (SNS) innervation of adipose tissue has been demonstrated in recent studies (14–16), and activation of the SNS prevents obesity by promoting brown fat thermogenesis and energy expenditure via the hypothalamic neuropeptide Y and norepinephrine. The SNS is an integral part of metabolism-related organs, but our understanding of the mechanisms by which the nervous system regulates the endocrine system to affect obesity is still lacking. understanding.

Activation of transient receptor potential vanilloid subtype 1 (TRPV1) sustains centrally regulated thermogenesis in peripheral tissues. TRPV1 is a nonselective cation channel (17). TRPV1 has a tetrameric structure consisting of three parts, the N-terminus and C-terminus located intracellularly, and six transmembrane regions (S1–S6) (18–20), with the N-terminal end playing a role in the activation of the channel. Early studies of TRPV1 focused on thermal and inflammatory pain transmission, and recent studies have revealed that TRPV1 also plays an important role in the regulation of tissue energy metabolism. A study of TRPV1 involvement in white adipose tissue (WAT) browning revealed (21) that the gene levels of TRPV1, silent message regulator 1 (Sirt1), and uncoupling protein-1 (UCP1) were suppressed in high-fat diet-fed mice, whereas capsaicin-fed mice presented a reversal of the expression levels of all these genes. Increasing evidence suggests that TRPV1 plays a key role in the regulation of body weight and lipid metabolism and is therefore considered a potential target for the treatment of obesity (22–26).

Given the increasing incidence of obesity annually, the resulting complications place heavy psychological and economic pressure on patients. Therefore, revealing the neuroendocrine regulatory mechanism of TRPV1 in obesity is particularly important. This paper reveals the core mechanism of TRPV1 in the endocrine system and the central nervous system of obese patients by reviewing previous studies on obesity and TRPV1 to provide a theoretical basis for stopping or even reversing obesity.

## 2 The potential role of TRPV1 in obesity

### 2.1 Neuromodulatory mechanisms of TRPV1

#### 2.1.1 Regulation of feeding behavior and energy metabolism by TRPV1 activation in the central nervous system

The TRPV1 protein in the central nervous system (CNS) plays an important role in the regulation of feeding behavior. TRPV1-positive neurons are widely distributed in the CNS (27–30), especially in the hypothalamus and nucleus tractus solitarius (NTS), which are closely related to food intake and energy expenditure (31, 32). TRPV1 expression in the hypothalamus of high-fat diet (HFD)-fed mice was significantly downregulated (33), whereas capsaicin restored its expression level, and activation of TRPV1 was able to increase energy expenditure and reduce body weight.

**Figure 1:**
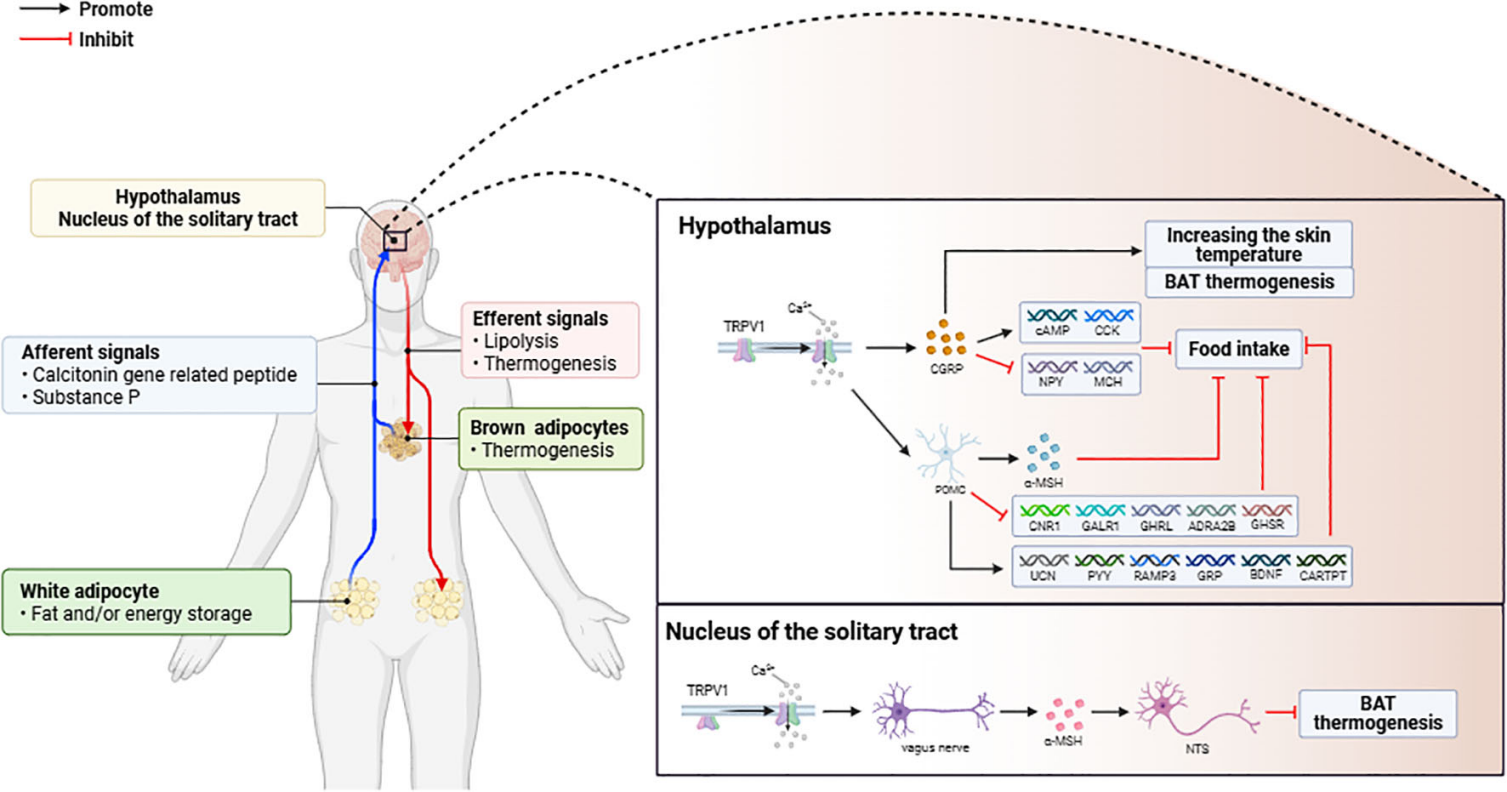
(1) Activation of TRPV1 in adipose tissue triggers the release of CGRP and SP, which mediate signal transduction in the central nervous system to regulate fat metabolism. (2) Activation of TRPV1 in the central nervous system (CNS) modulates the expression of appetite and adipose thermogenesis-related genes, leading to weight loss through increased energy expenditure.

The function of TRPV1 is related to its distribution. In the hypothalamus, TRPV1-positive neurons coexpress a variety of neuropeptides [including calcitonin gene-related peptide (CGRP), NPY, and substance P (SP)] to regulate peripheral thermogenesis and dietary intake (33). TRPV1 activation induces Ca^2+^ influx, which may be crucial for the release and function of CGRP (34, 35), and CGRP may inhibit food intake by increasing cyclic adenosine monophosphate (cAMP) and cholecystokinin (CCK) expression in the hypothalamus, downregulating the expression of appetite-inducing neuropeptides (NPY and MCH), and increasing skin temperature and brown adipose tissue (BAT) thermogenesis (34, 36). In POMC neurons, which regulate appetite and satiety, TRPV1 activation releases α-melanocyte-stimulating hormone (α-MSH) to act on satiety centers, leading to a reduction in appetite (37), and this process is TRPV1 dependent. In terms of hypothalamic gene expression profiles in HFD-fed mice, TRPV1 activation upregulates the expression of satiety-related neuropeptide genes (e.g., UCN, PYY, RAMP3, GRP, BDNF, and CARTPT) and downregulates the expression of appetite-stimulating genes (e.g., CNR1, GALR1, GHRL, ADRA2B, and GHSR), reducing food intake and body weight (33, 38–41). (**Figure 1**).

Activation of TRPV1 in the solitary tract nucleus (NTS) inhibits BAT activation in HFD-fed rats. The levels of linoleic acid metabolites (LAs) are elevated in the NTS of HFD-fed rats, and these metabolites can act as endogenous TRPV1 activators (42). The activation of TRPV1 at the afferent terminals of the vagus nerve induced the release of glutamate to increase the activity of the neurons in the NTS, which in turn inhibited the sympathetic excitatory neurons of the BAT, forming a pathway to inhibit brown adipose tissue (BAT) thermogenesis and energy expenditure. pathway. Therefore, the energy metabolism regulatory function of TRPV1 is spatially inhibitory (**Figure 1**).

#### 2.1.2 Central regulation of tissue energy metabolism is dependent on activation of TRPV1 in peripheral sensory nerves

Peripheral sensory nerves are involved in regulating adipose tissue thermogenesis and WAT browning processes. Studies have shown that BAT-specific denervation in rats is associated with increased body weight; decreased resting metabolic rates; decreased BAT mass; decreased adipocyte and mitochondrial numbers; downregulated UCP1 protein expression; and decreased core body temperature (43–45). In contrast, both unilateral and bilateral ablation of subcutaneous WAT in mice upregulated the expression of thermogenic genes and was accompanied by beige adipocyte formation (8). These findings suggest that neuromodulation is necessary to maintain the homeostasis of fat energy metabolism.

The regulation of energy metabolism in adipose tissue is dependent on neuropeptide secretion following TRPV1 activation. Mammalian adipose tissue function is regulated by the peripheral nervous system (8), and the activation of TRPV1 in BAT and WAT sensory neurons results in the expression of the neuropeptides CGRP and SP (46, 47), which transmit information from adipose tissue to the central nervous system (hypothalamus, solitary tract nucleus, etc.) through synaptic links between neurons, and the removal of sensory nerves of the adipose tissue results in compensatory hyperplasia, further demonstrating the involvement of sensory signaling in systemic adipose homeostasis (48, 49). Tracing of sympathetic nerves and sensory nerves innervating the BAT revealed colocalization in the central nervous system (hypothalamus, solitary tract nucleus, and other brain regions) (49, 50), suggesting that there is a direct interaction between sympathetic and sensory signals at the center. Thus, sympathetic and sensory nerves synergistically regulate fat metabolism through a bidirectional loop, with sympathetic nerves dominating lipolysis and thermogenesis and sensory nerves feeding back on fat status to modulate sympathetic output, a mechanism that is functionally specific in WAT and BAT but shares some central nodes.

In addition, the neuropeptides CGRP and SP released upon sensory nerve activation exert regulatory effects on adipose tissue metabolism. Previous studies have shown that CGRP has hormonal effects as a neuropeptide (51). Lipid metabolism regulation by CGRP may occur through changes in plasma catecholamine, cortisol, glucagon, insulin, lactate, and adipokine levels, as well as in the blood supply of adipose tissue (36, 52–60). SP upregulates neurokinin 1 receptor (NK1R) mRNA and protein expression levels in human preadipocytes (61). SP also promotes lipolysis in 3T3L1 adipocytes, blocks insulin-mediated fatty acid uptake, and inhibits the accumulation of lipid droplets during differentiation (62). In contrast, high-fat diet-induced weight gain was inhibited in NK1R-/- mice, circulating levels of insulin and leptin were reduced, and insulin-dependent glucose uptake was improved (63). Thus, neuropeptides secreted upon the activation of sensory nerve TRPV1 not only play a role in central regulation but also play a role in regulating local adipose tissue (**Figure 1**).

### 2.2 Endocrine regulatory mechanisms of TRPV1

#### 2.2.1 Adipose tissue TRPV1 activation promotes mitochondrial oxidation

Adipose tissue is an important part of the human body. Owing to its structure and function, it can be divided into WAT, which is responsible for storing fat, maintaining body temperature and regulating metabolism throughout the body, and BAT, which generates a large amount of heat energy through the catabolism and oxidation of lipids and helps to maintain body temperature.

**Figure 2:**
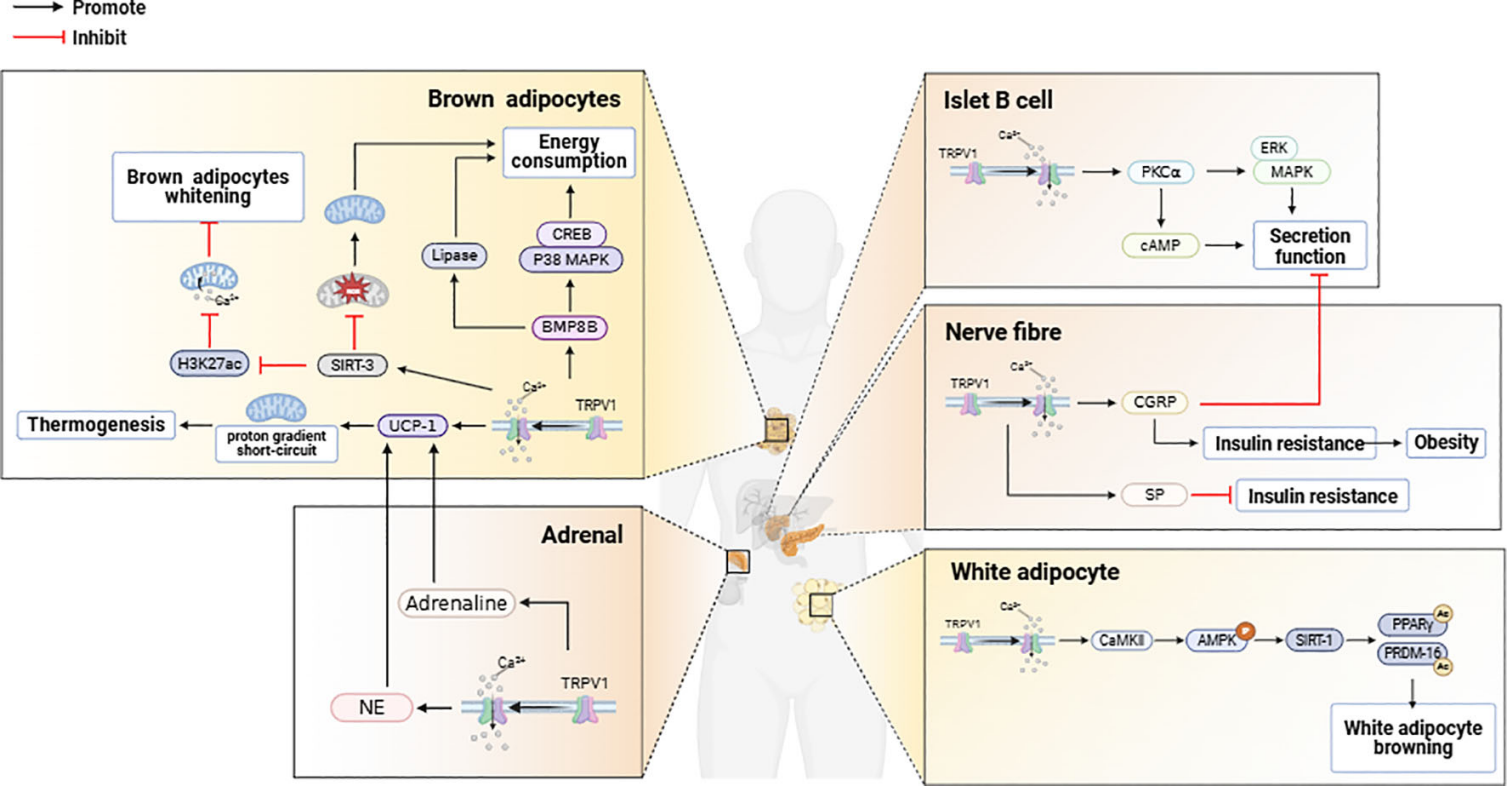
TRPV1 activation in brown adipose tissue, white adipose tissue, pancreas, and adrenal glands exerts regulatory effects on energy metabolism.

Adipose tissue TRPV1 activation to promote mitochondrial energy metabolism is required for WAT browning. WAT browning has been used as a novel strategy to improve metabolic health (6), and WAT browning is able to inhibit energy intake-induced weight loss by triggering thermogenesis to promote energy expenditure (40, 64, 65). Upon the activation of WAT-expressed TRPV1 (66, 67), the intracellular Ca^2+^ concentration increases, and the activation of calmodulin kinase II (CaMKII) causes the phosphorylation of AMP protein kinase (AMPK), leading to the activation of sirtuin 1 (SIRT-1), which serves as a sensor of cellular metabolism and energy utilization (68, 69), the activation of which leads to the deacetylation of PPARγ and PRDM-16, both of which promote WAT browning (70, 71). With the activation of TRPV1, the expression of UCP-1 and bone morphogenetic protein 8B (BMP8B) is upregulated. UCP-1 is localized on the inner mitochondrial membrane, and when activated, it short-circuits the mitochondrial proton gradient, thus promoting thermogenesis (66, 72). By increasing p38 MAPK/CREB signaling and adiponectin activity, BMP8B enhances the sensitivity of BAT to NE to promote energy expenditure (73). Upon activation of TRPV1, the mitochondrial deacetylase SIRT-3 is activated, leading to a decrease in ROS production (74) and an increase in energy metabolism due to increased mitochondrial activity. SIRT-3 was also able to downregulate the expression of H3K27ac on the mitochondrial calcium unidirectional transporter (MCU) promoter via an AMPK-dependent pathway, which inhibited mitochondrial calcium ion overload to prevent BAT whitening. The expression of the adipogenic regulators Pparγ2 and PPARγ coactivator 1α (Pgc-1α) in BAT is also upregulated upon activation of TRPV1 (75). Pparγ2 promotes transcriptional cascades involved in adipocyte function (76, 77), whereas Pgc-1α stimulates mitochondrial biogenesis as well as BAT cell function, including transcriptional activation of Ucp1 (78). Mitochondrial homeostasis in BAT is critical for maintaining BAT thermogenesis, and mitochondrial Ca^2+^ regulates the activity of essential metabolic enzymes and transporter proteins (79). TRPV1 maintains mitochondrial Ca^2+^ homeostasis in BAT by repressing the expression of the ion channel protein LETM1 (80). When genes regulating TRPV1 expression are knocked down, the expression of UCP1 and LETM1 tends to increase, leading to disturbances in mitochondrial Ca^2+^ homeostasis in BAT and aggravating obesity (**Figure 2**).

Studies have shown that TRPV1 activation in murine and human adipose precursor cells upregulates the cytoplasmic receptor responsible for calcium cycling (α1-AR), the calcium-sensing enzyme (CaMKII), and mitochondrial calcium transporters (VDAC and MCU), leading to increased intracellular Ca²^+^ concentrations, which suppress adipogenesis in adipose precursor cells and promote UCP1-dependent thermogenesis (81, 82). Following the activation of TRPV1, the mRNA levels of hormone-sensitive lipase (HSL), carnitine palmitoyltransferase Ia (CPT-Ia), which is a rate-limiting enzyme in mitochondrial fatty acid oxidation, and uncoupling protein 2 (UCP2) are increased (83–86). This results in increased lipolysis in adipocytes and a reduction in the intracellular lipid content (**Figure 2**).

#### 2.2.2 Pancreatic β-cell TRPV1 activation regulates pancreatic function

The pancreas is an important visceral organ in the regulation of glucose metabolism, and insulin, an anabolic hormone, is synthesized and secreted by pancreatic beta cells (87). In peripheral tissues, insulin promotes glucose uptake in adipose tissue and inhibits lipolysis, promoting fat storage in adipocytes (88, 89), and in the central nervous system, insulin acts as an appetite suppressant to decrease food intake and body weight (90).

TRPV1 activation in pancreatic β-cells increases insulin secretion. Previous studies have shown that TRPV1 affects pancreatic function and insulin secretion in both humans and animals (91), and the activation of TRPV1 expressed on pancreatic β-cells by calcium influx increases insulin secretion, a process that involves the regulation of protein kinase C alpha (PKC alpha) and cyclic adenosine monophosphate cAMP (92, 93). In addition, TRPV1 is coexpressed with CGRP in pancreatic nerve fibers (94), and inhibition of TRPV1 signaling decreases CGRP secretion, thereby increasing insulin secretion. Insulin sensitizes TRPV1 in sensory nerve endings (95, 96), and TRPV1-activated neurons regulate pancreatic β-cell function through the release of neuropeptides such as SP and CGRP (97–100), where an increase in CGRP secretion decreases insulin release from pancreatic β-cells (98). Sustained high levels of circulating CGRP can lead to insulin resistance and obesity, whereas increased SP secretion can alleviate insulin resistance (97). Furthermore, TRPV1 is coexpressed with CCK-sensitive vagal afferent neurons. Studies have revealed that TRPV1 activation-induced calcium influx enhances the responsiveness of vagal afferent neurons to CCK, leading to increased vagal signaling, which regulates pancreatic secretory function to maintain metabolic homeostasis. This mechanism may involve low-affinity CCK binding to CCK1 receptors (CCK1Rs), triggering downstream signaling (e.g., Gq proteins or β-arrestin) (101). Recent studies have indicated that TRPV1 activation may contribute to β-cell dysfunction and acute pancreatitis, whereas TRPV1 antagonists restore SP/CGRP expression levels, increase the islet area, reduce pancreatic β-cell vacuolization, decrease proinflammatory cytokine (TNF-α, IL-1β) release, and increase anti-inflammatory IL-10 secretion (102). This process may involve the modulation of the JAK2-STAT3 signaling pathway (103). (**Figure 2**).

#### 2.2.3 Regulation of energy metabolism by adrenal TRPV1 activation

The adrenal gland is composed of the cortex and medulla and is involved in the regulation of energy metabolism as an endocrine gland (104). The adrenal cortex synthesizes and secretes steroids, whereas the medulla produces catecholamines and neuropeptides (105–107). Epinephrine and norepinephrine maintain energy production through lipolysis and ketogenesis during hypoglycemia and malnutrition (108–110). Catecholamine binding stimulates β3-adrenergic receptors, leading to increased intracellular cAMP concentrations and the activation of cyclic AMP-dependent protein kinase A (PKA), which leads to the phosphorylation and activation of hormone-sensitive lipase (HSL) to increase adipocyte lipolysis (111). In addition, catecholamine stimulation of α2-adrenergic receptors inhibits lipolysis (112). These adrenergic responses depend on the density of these two receptor families, their relative affinities, and the location and amount of adipose tissue (113). Obesity may alter the sensitivity of alpha- and beta-adrenergic receptors in adipose tissue, thereby altering the effects of catecholamines on lipolytic processes and increasing fat storage (114, 115). Glucocorticoids (GCS) plays an important role in the regulation of metabolic homeostasis (116). Chronical elevation of GCs can alter body fat distribution, increasing visceral obesity and metabolic abnormalities (117, 118). (**Figure 2**).

TRPV1 plays an important role in metabolic pathways related to energy homeostasis and insulin signaling. In the rat adrenal gland, there are TRPV1-positive nerve fibers; in particular, TRPV1-positive fibers are observed in the adrenal tegument, cortex and medulla (119), where 35% of the medullary cells and 20% of the cortical cells express this cation channel (120). The colocalization of TRPV1 in the adrenal medulla with CGRP, which is stored in sensory nerve endings, is associated with pain perception, inflammatory responses and increased catecholamine secretion in the adrenal medulla (121, 122). Catecholamines serve as an important class of neurotransmitters and hormones, including epinephrine, norepinephrine, and dopamine, which play key roles in the regulation of the nervous system, cardiovascular system, and energy metabolism (123). Sensory nerves expressing TRPV1 promote energy expenditure by activating sympathetic nerves and promoting noradrenaline secretion (124).

TRPV1 channels may be activated by acidic contents released by adrenal medullary cells (125), and synergistically with the activation of P2X3 receptors, they lead to the secretion of catecholamine hormones by the adrenal medulla. After the activation of TRPV1, the secretion of norepinephrine is stimulated via β2 adrenergic receptors and β3 adrenergic receptors, which increase the expression of UCP1 in BAT. This leads to a reduction in visceral fat content in obese rats induced by a high-fat diet (78, 126, 127). BAT plays a major role in diet-induced thermogenesis, and UCP1 is thought to be a key thermogenic regulator of BAT (78). Previous studies have demonstrated that the activation of TPPV1 enhances the secretion of epinephrine (128, 129), which increases energy expenditure and thermogenesis through the activation of adrenergic receptors (130, 131), whereas in adrenal-depleted rats, thermogenesis resulting from TPPV1 activation is markedly attenuated (132). In addition, adrenergic receptor activation upregulated UCP1 expression in BAT, increasing WAT browning and BAT thermogenesis (78, 133–140). In addition, TRPV1 activation in adrenocortical cells leads to an increase in intracellular calcium ion levels, which in turn inhibits GC secretion, reducing the occurrence of visceral obesity (141). (**Figure 2**).

## 3 TRPV1-targeted therapy

At this stage, the treatment strategy for obesity still emphasizes lifestyle changes, such as avoiding a sedentary lifestyle, proper exercise and a balanced diet. However, the incidence of obesity remains high. Previous studies on TRPV1-targeted therapy have focused mostly on relieving discomfort, such as pain and itching caused by the disease (142, 143), and with increasing research, TRPV1 has been recognized as a potential target with preventive effects against obesity (25). TRPV1 plays an important role in the regulation of pain sensation, body heat production and energy metabolism. In previous clinical trials, the use of TRPV1 antagonists increased the thermal threshold and increased the risk of burns, which makes it difficult for related drugs to enter clinical phase III trials (144). Most TRPV1 antagonists used in clinical studies involve varying degrees of body temperature elevation in experimental subjects. Most TRPV1 antagonists have shown different degrees of temperature elevation in subjects in clinical studies (144, 145), and their molecular mechanisms are still poorly understood. Moreover, TRPV1 agonists such as capsaicin have been shown to induce a decrease in body temperature in animal studies (146). In a related study, resveratrol significantly improved the discomfort induced by TRPV1 agonists (147), suggesting that these side effects could be avoided or even eliminated. A new generation of TRPV1 antagonists has been reported to have a weaker effect on body temperature in clinical trials (148). The clinically safe TRPV1 antagonist XEN-D0501 is currently under development as an oral drug for overactive bladder (148); undoubtedly, these findings provide valuable clinical data for the development of TRPV1-targeted drugs to treat obesity.

## 4 Conclusion

Obesity is a metabolic syndrome characterized by excessive accumulation of fat in the body due to an imbalance between energy intake and expenditure. In previous studies, conventional treatments for obesity included lifestyle interventions (such as dietary restrictions and physical exercise), bariatric surgery, and drug therapy (149). At present, the drugs commonly used in clinical practice for treating obesity all have certain efficacy, but most of them cause various side effects (150). In this review, by summarizing the mechanism of the role of TRPV1 in the endocrine system and the central nervous system under conditions of obesity, we found that TRPV1 plays an important role in the occurrence and development of obesity and participates in the processes of energy intake and consumption. Strict control of energy homeostasis is crucial for maintaining a healthy weight or for helping with weight loss by expending more energy than is consumed. TRPV1 is involved in energy homeostasis, regulating both food intake and energy expenditure. TRPV1 may affect appetite by controlling the levels of appetite hormones, and it can also increase energy expenditure by generating heat.

TRPV1, a nonselective cation channel, is also an important receptor. The acute activation of TRPV1 leads to conformational changes in TRPV1, causing the opening of the TRPV1 channel, resulting in a large influx of Ca²^+^ and Na^+^, triggering cell depolarization. The influx of Ca²^+^ prompts sensory nerve endings to release CGRP and SP, mediating neurogenic inflammation (vasodilation, plasma extravasation). Chronic activation of TRPV1 (such as long-term exposure to capsaicin or inflammatory stimuli) causes changes in the phosphorylation state of TRPV1, leading to channel desensitization. Research has shown that normal rats typically lose weight after long-term capsaicin desensitization, and this process is associated with a reduction in fat accumulation (81, 151). Long-term activation of TRPV1 can also increase energy expenditure by enhancing the thermogenic capacity of brown adipose tissue and promoting the browning of white adipose tissue (67, 152). After capsaicin activates TRPV1, it increases the abundance of beneficial bacteria in the intestine, promoting the production of bile acids (BAs) and short-chain fatty acids (SCFAs) and increasing the secretion of glucagon-like peptide-1 (GLP-1) and peptide YY (PYY), thereby increasing satiety, reducing food intake, and influencing energy metabolism and inflammatory responses (153, 154). Furthermore, obesity, a form of chronic inflammation, increases the circulating levels of fat and inflammatory cytokines (155, 156). The activation of TRPV1 is regulated by various inflammatory mediators, including nerve growth factor (NGF), prostaglandins (PGs), bradykinin (BK), leukotrienes (LTB4), etc. These mediators increase the sensitivity of TRPV1 through different signaling pathways (such as PKA, PKC, and MAPK), thereby activating TRPV1 (157, 158). When TRPV1 is activated, it regulates downstream pathways and participates in the regulation of obesity. The acute activation of TRPV1 serves as a warning system for the body to avoid harm (such as burns), whereas chronic activation induces adaptive protection (such as maintaining vascular homeostasis, anti-inflammatory desensitization, and metabolic regulation). Therefore, chronic activation of TRPV1 has broader application potential for metabolic diseases.

Although a large body of evidence points to a relationship between the development of obesity and TRPV1, the relationship between the two is still controversial in some studies. For example, TRPV1 knockout mice have been reported to lose body weight (22), but other studies have shown that TRPV1 knockout mice are not obese at young ages. However, the weight of TRPV1 knockout mice increases significantly during aging (159). These findings suggest that the regulatory effect of TRPV1 on obesity may be age dependent, and a similar relationship was also shown in healthy subjects (160). Therefore, age should be considered a potential influencing factor in the study of TRPV1 and obesity. Nevertheless, aberrant TRPV1 activation and expression may contribute to the onset and development of obesity. Therefore, TRPV1 may be a target for the treatment of weight loss disorders, and finding a drug or stimulation method (mechanical stimulation or temperature stimulation) that can treat obesity by acting on TRPV1 will be our next research direction.
